# A longitudinal cross-sectional analysis of substance use treatment trends for individuals experiencing homelessness, criminal justice involvement, both, or neither - United States, 2006-2018

**DOI:** 10.1016/j.lana.2021.100174

**Published:** 2022-01-06

**Authors:** Riley D. Shearer, Nathan D. Shippee, Kathrine Diaz Vickery, Maria A. Stevens, Tyler N.A. Winkelman

**Affiliations:** a Department of Health Policy and Management, School of Public Health, University of Minnesota, Minneapolis, MN, USA; b Health, Homelessness, and Criminal Justice Lab, Hennepin Healthcare Research Institute, Minneapolis, MN, USA; c General Internal Medicine, Department of Medicine, Hennepin Healthcare, Minneapolis, MN, USA; d Department of Health Policy and Management, Gillings School of Global Public Health, University of North Carolina at Chapel Hill, Chapel Hill, NC, USA

**Keywords:** homeless, criminal justice, methamphetamine, polysubstance, opioids, treatment

## Abstract

**Background:**

Individuals experiencing homelessness or criminal justice involvement (CJI) have higher rates of substance use than the general public. Despite documented barriers to accessing treatment, few studies have compared substance use treatment patterns between these groups.

**Methods:**

This paper uses data from the Treatment Episode Dataset-Admissions between 2006 to 2018 to describe characteristics and trends in substance use treatment admissions indicating homelessness (n=2,524,413), CJI (4,764,750), both (509,902), or neither (8,950,797) in the United States. We used multivariable logistic regression to examine trends independent of demographic differences between groups.

**Findings:**

Between 2006 and 2018, the proportion of treatment admissions related to heroin increased across all groups. Methamphetamine-related admissions rose substantially for individuals experiencing homelessness, CJI, or both. By 2018, 27·8% (95% CI: 27·4-28·2%) of admissions for individuals experiencing both were methamphetamine-related and 16·7% (95% CI: 16·3-17·0%) were heroin-related. Conversely, among individuals experiencing neither, 7·5% (95% CI: 7·4-7·5%) of admissions were methamphetamine-related and 33·6% (95% CI: 33·4-33·7%) were heroin-related. Individuals experiencing both homelessness and CJI received lower rates of medications for opioid use disorder (OUD) (8·3%; 95% CI: 8·2-8·3%) compared to individuals experiencing neither (36·4%; 95% CI: 36·4-36·4%).

**Interpretation:**

Community treatment facilities should be supported to provide medications for OUD and accommodate rising rates of methamphetamine and polysubstance-related treatment admissions in populations experiencing complex social drivers of health such as homelessness, CJI, or both.

**Funding:**

National Institute of General Medical Sciences and National Institute of Diabetes and Digestive and Kidney Diseases.

**Research in context**

**Evidence before this study**

There is a large body of evidence highlighting the public health consequences of substance use among individuals experiencing homelessness or criminal justice involvement (CJI). We searched PubMed and Google Scholar from database inception through October 31, 2020 using combinations of the search terms “substance use”, “substance abuse”, “substance dependence”, “treatment”, “trend”, “incarceration”, “criminal justice involvement”, “homeless”, and “housing instability”. We identified one article which described trends in substances leading to treatment admission for individuals experiencing homelessness in the United States (US) which found the proportion of treatment admissions for methamphetamine and opiates increased between 2005 and 2015. One article describing correlates and trends in substance use disorders among individuals with CJI found no change between 2002 and 2014, but did not examine potential changes in treatment admissions. A cross sectional study of individuals experiencing homelessness in three Canadian cities found that substance use varied significantly between individuals with and without CJI. To our knowledge no study has described trends in substances leading to treatment admission or differences in treatment characteristics for individuals experiencing homelessness, CJI, or both. Surveillance data from the US suggests that substances involved in drug overdose deaths have shifted over the past decade. Additionally, previous studies have demonstrated homelessness and CJI as risk factors for substance use, but it remains unknown how substance use treatment admissions have changed for these groups or how these trends compare to the general population.

**Added value of this study**

Using 2006-2018 data for all substance use treatment admissions to facilities in the US receiving public funding, we identified the proportion of treatment admissions due to methamphetamine, heroin, alcohol, cocaine or crack, and other opioids. We compared treatment characteristics and trends in substances leading to treatment between admissions for patients experiencing homelessness, CJI, both, or neither. Compared to admissions for individuals experiencing neither, admissions indicating homelessness, CJI, or both were more likely to be for methamphetamine and less likely to be for heroin or other opioids. Treatment for individuals experiencing homelessness is likely of lower quality as indicated by a higher proportion of admissions to a detoxification facility and lower rates of medications for opioid use disorder.

**Implications of all the available evidence**

Our findings describe important differences in trends of substance-specific treatment admissions among individuals experiencing homelessness, CJI, or both and contribute to the growing body of evidence that individuals experiencing homelessness, CJI, or both receive lower rates of medications for opioid use disorder. These findings can be used to guide federal and state government responses to rising overdose deaths in the US. Equitable allocation of resources should ensure that 1) community facilities are equipped to provide treatment for substances used by the most at risk populations and 2) treatment quality is not lower for individuals experiencing homelessness, CJI, or both.

Alt-text: Unlabelled box

## Introduction

Overdose-related deaths and hospitalizations continue to increase in the United States (US) and are the leading cause of death among individuals experiencing homelessness, criminal justice involvement (CJI), or both.^1^, ^2^, ^3^ Barriers to substance use treatment experienced by the general population (e.g., cost, motivation, or knowing where to receive treatment) are even more prevalent for people experiencing homelessness, CJI, or both.^4^, ^5^, ^6^ Substance use treatment in the US is fragmented across public and private payers and is minimally connected to the traditional health care system.^7^ This creates barriers for all individuals with SUD, which are further compounded for people experiencing homelessness or CJI by poverty, a history of trauma, stigma, discrimination in healthcare settings, and high rates of comorbid mental health diagnoses.^4^^,^^5^^,^^8^, ^9^, ^10^, ^11^, ^12^, ^13^ Individuals experiencing homelessness face structural barriers such as transportation, fragmented healthcare systems, lack of health insurance, food, and stable housing, which may impede access to substance use treatment.^6^^,^^10^^,^^14^^,^^15^ Individuals with CJI may experience unique structural barriers such as lack of health insurance and disenrollment from Medicaid, probation or parole constraints, emotional distress from transitional challenges, and limited availability of medication for opioid use disorder (OUD) in the justice system.^16^, ^17^, ^18^, ^19^ Additionally, gender differences exist in the pathways to homelessness and/or CJI as well as barriers to treatment such as stigma and a history of trauma.^5^^,^^20^ The barriers to substance use treatment for individuals experiencing homelessness, CJI, or both are particularly harmful because these groups have substantially higher rates of substance use disorders than the general population, including higher rates of cocaine and methamphetamine use.^12^^,^^21^^,^^22^ Additionally, both homelessness and CJI are independently associated with human immunodeficiency virus (HIV) risk factors including injection drug use and sharing syringes, further increasing the importance of treatment for these populations.^23^^,^^24^

Despite unique barriers to substance use treatment and higher rates of substance use among individuals experiencing homelessness, CJI, or both compared with the general population, differences in substance use treatment utilization remain poorly defined. One study highlighted substance use treatment admission trends among people experiencing homelessness, but it is now several years old and did not compare them to individuals with CJI or the general population.^25^ Another study examined substance use treatment for individuals experiencing both homelessness and CJI, but relied on a relatively small and geographically limited sample and did not examine individuals with homelessness or CJI alone or the general population.^26^ The Affordable Care Act (ACA), which was the largest expansion of health insurance coverage in the US among low-income populations in 50 years, increased access to substance use treatment and improved the rate of medications for OUD among individuals experiencing CJI.^27^^,^^28^ The key provisions of the ACA went into effect in 2014, but continue to evolve due to ongoing political and judicial deliberations.^29^ The dynamic nature of substance use crisis has been observed in the general population as specific substances led to different peaks over time. However, to our knowledge, a comprehensive examination of treatment trends across individuals experiencing homelessness, CJI, or both in the US does not exist. An analysis of trends in substance use treatment between the general population and these groups can illuminate unique treatment patterns to inform investment in substance use treatment that meets the needs of already marginalized populations. While many social factors intertwine substance use and treatment utilization, we focus on homelessness and CJI in this paper as they are two sectors, that represent opportunities for targeted interventions because they are administered by specific funding sources and policies in the US.

In this paper we utilized US substance use treatment data from 2006-2018, to examine treatment trends among individuals experiencing homelessness, CJI, or both and the general population. We characterized trends in the specific substances leading to treatment for each group. Among all substance use treatment admissions, we compared treatment setting between groups. Among treatment admissions indicating opioid use, we compared the receipt of medications for OUD between groups. Given recent data that showed high levels of unstable housing and CJI among people who used any methamphetamine, we hypothesized that a higher proportion of treatment admissions indicating homelessness, CJI, or both would be related to methamphetamine relative to the general population.^30^

## Methods

### Data source

We used substance use treatment data from the Treatment Episode Data Set - Admissions (TEDS-A), a publicly available data set from the Substance Abuse and Mental Health Services Administration (SAMHSA).^31^ Substance use treatment centers in the US that receive public funding are required to report data on treatment admissions to their respective states, which in turn report data to SAMHSA. Treatment centers are required to report on admissions that were funded through federal sources, though many centers that receive any public funding report on all admissions, regardless of funding source. The scope of treatment centers required to report admission data varies slightly between states.^32^ Approximately 60% of states reported on all admissions to eligible facilities, while other states reported admissions that were publicly funded. The following states did not report admissions in all years: Georgia (2016-2018), Oregon (2015-2018), South Carolina (2014-2015), District of Columbia (2006 & 2009), Mississippi (2009), Alabama (2007), and Alaska (2006).^33^ Data include patient, substance use, and facility characteristics. Because TEDS-A contains data at the treatment admission level rather than individual level, some individuals may be represented multiple times. We included data on all treatment admissions reported to SAMHSA between 2006-2018 for individuals aged 18 and older.

### Exposure groups

We used living arrangement and referral source to define four mutually exclusive groups: individuals experiencing homelessness (but not CJI), CJI (but not homelessness), both homelessness and CJI, and neither homelessness nor CJI. In TEDS-A, living arrangement at the time of admission is reported as homeless, dependently housed, or independently housed. Homeless is classified as having no fixed address or residing in a homeless shelter. Dependent housing is comprised of residential institutions, group homes, halfway houses, or a minor living with guardians. Because this classification includes both stable and unstable living arrangements, we excluded treatment admissions of dependently housed individuals in our primary analysis. In the US, there are a variety of referral sources to substance use treatment including: individual (self, family member, or friend), healthcare professional, school, employer, community (including religious organizations, government agencies, and self-help groups), and the criminal justice system. We defined individuals as having CJI if the referral source included a police official, judge, prosecutor, probation officer or other person affiliated with the criminal justice system, including a court for DWI/DUI. We excluded admissions for individuals aged 17 and younger because the experience in the US criminal justice system may differ between minors and adults.

### Admissions by substance use type

TEDS-A includes information about the primary, secondary, and tertiary substance use leading to treatment admission. Treatment facilities list a substance if it led to admission, but this does not necessarily reflect the Diagnostic and Statistical Manual of Mental Disorders definitions for substance use disorders. We analyzed admissions that were primarily due to alcohol, cocaine or crack, heroin, other opiates (non-prescription methadone, prescription opioids, and synthetic opioids), or methamphetamine (including other amphetamines which accounted for 5% of the methamphetamine-related admissions). We also examined trends in methamphetamine and heroin co-use, given its association with increased morbidity and mortality and previously identified high rates of co-use among individuals experiencing homelessness and/or CJI.^34^ We defined methamphetamine and heroin co-use as a treatment admission indicating both heroin and methamphetamine as the primary, secondary or tertiary substances leading to admission.

### Sociodemographic controls

We assessed age in years (18-24, 25-29, 30-39, 40-49, and 50+), sex (male and female), education (less than high school, high school or GED, and some college), race (White, Black, Hispanic, American Indian/Native Hawaiian or Alaskan, and Other), employment (employed and unemployed), and US census region (Northeast, Midwest, South, and West) among the groups indicating homelessness only, CJI only, both, and neither.^35^ These covariates have been shown to be associated with the prevalence of substance use and treatment characteristics in previous research.^36^, ^37^, ^38^, ^39^ Because treatment characteristics vary substantially by facility type (detoxification, residential, or ambulatory), we also measured what proportion of admissions were to each facility type.^40^ Detoxification facilities can be freestanding or in a hospital and provide a setting for safe withdrawal, residential facilities included short and long-term facilities as well as inpatient hospital treatment (other than detoxification), ambulatory facilities provide outpatient care. We adjusted for these measures in all analyses. Fewer than 5% of admissions were missing data for referral source or sociodemographic controls, which we excluded using case-wise deletion.

### Treatment characteristics

To assess treatment characteristics, we analyzed the facility type and whether an admission had received prior substance use treatment. Additionally, we measured the proportion of admissions indicating heroin or other opioids that included medications for OUD in the treatment plan.

### Statistical analysis

First, we compared sociodemographic characteristics and treatment facility type for admissions among individuals experiencing homelessness, CJI, both, or neither. We then tabulated the number of treatment admissions for each group by year to assess trends in treatment volume. Next, in a repeated cross-sectional analysis, we used multiple logistic regressions, adjusting for sociodemographic characteristics and treatment facility type, and post estimation predictive margins to calculate the proportion of treatment admissions due to alcohol, cocaine or crack, heroin, other opiates, and methamphetamine among the four groups for each year. To test the significance of time trends, we then ran the regression models with the year specified as a continuous variable and assessed whether the coefficient for the linear time trend variable was significant. To assess differences in trends by sex, we repeated the analyses of trends in treatment volume and proportion of treatment admissions due to specific substances stratified by male and female patients. We then calculated the proportion of treatment admissions meeting our criteria for both methamphetamine and heroin use.

We used multiple logistic regressions, followed by postestimation predictive margins to calculate rates of prior treatment, and planned medication for OUD use among the four groups. First, we estimated these models adjusting for sociodemographic characteristics only, then we re-estimated them with both sociodemographic characteristics and treatment facility type as co-variates. We conducted pairwise comparisons (between each exposure group and all other groups, 6 in total for each outcome) to assess whether differences in the estimates of treatment characteristics were statistically significant. In a sensitivity analysis, we included treatments of individuals in dependent housing for comparison to treatments for individuals experiencing homelessness. To assess whether including multiple treatment episodes for one person substantially affected our findings, we conducted an additional sensitivity analysis and restricted the sample to treatment episodes for individuals with no prior treatment. We used Stata 17.0 for all analyses and considered p<0·05 to be statistically significant.

### Ethical consideration

Institutional Review Board approval and informed consent were not required because all data are publicly available from SAMHSA and deidentified.

### Role of funding source

The funder had no role in study design, data collection, analysis, interpretation, or writing of this report. The content is solely the responsibility of the authors and does not necessarily represent the official views of the National Institutes of Health.

## Results

### Study population

**Figure 1:**
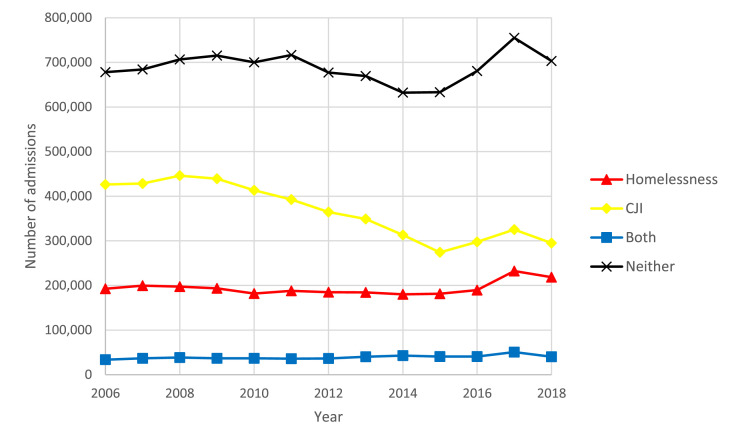
Trends in overall treatment admissions by year and group: homelessness, criminal justice involvement (CJI), both, and neither, 2006-2018

**Figure 2:**
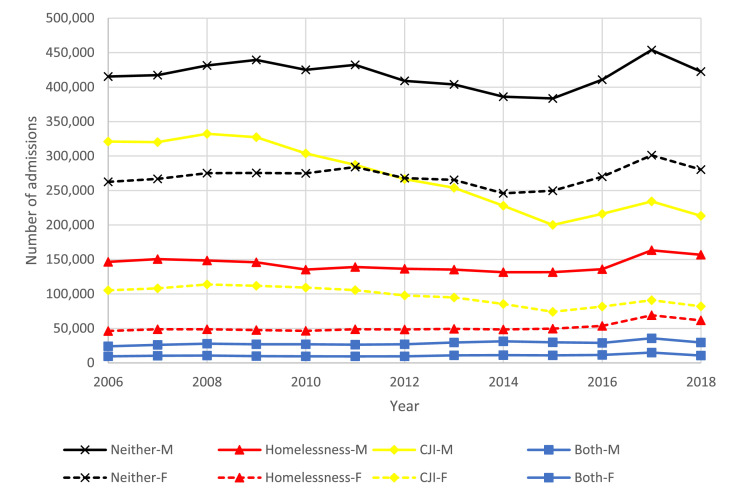
Trends in overall treatment admissions by year, sex, and group: homelessness, criminal justice involvement (CJI), both, and neither, 2006-2018

Between 2006 and 2018, we identified 17,779,599 unique treatment admissions that met our cohort specification for housing and age. We then excluded 327,825 (1·8%) admissions missing data for referral source. An additional 701,912 (3·9%) treatment admissions were excluded due to missing sociodemographic or treatment facility type data resulting in a final sample of 16,749,862 unique admissions. Among our sample, 2,524,413 (15·1%) reported homelessness only, 4,764,750 (28·4%) reported CJI only, 509,902 (3·0%) experienced both homelessness and CJI, and 8,950,797 (53·4%) experienced neither CJI nor homelessness. Between 2006 and 2018 the total number of treatment admissions decreased by 6% (1,330,635 in 2006 to 1,256,937 in 2018). This was driven by a 44% decrease in treatment admissions for individuals with CJI decreasing from 426,365 treatment admissions in 2006 to 295,187 in 2018. Treatment admissions for individuals experiencing homelessness, both, or neither rose 12%, 17%, and 4%, respectively (Figure 1). Trends in treatment admissions for men and women differed between 2006 and 2018. Admissions decreased more for men than women experiencing CJI (34% vs. 22% decrease) and increased more for women than men experiencing homelessness (33% vs. 7%). (Figure 2).

Compared with admissions for those experiencing neither homelessness nor CJI (60·7% male and 26·7% less than a high school education) admissions for individuals experiencing homelessness, CJI, or both were more likely to be male (73·6%, 73·6%, 72·7%, respectively) and have less than a high school education (32·5%, 29·1%, 34·5%, respectively) (Table 1). Admissions for those experiencing homelessness were on average older (52·4% over 40 years old) than admissions with CJI (30·6% over 40 years old) or neither (40·6% over 40 years old). Admissions for individuals experiencing homelessness, CJI, or both were more likely to be Black (27·1%, 19·2%, 19·5%, respectively) and less likely to be White (51·3%, 61·3%, 52·7%, respectively) than individuals experiencing neither (65·6% and 17·8%). Admissions for individuals experiencing homelessness had the highest rate of unemployment (94·9%) while individuals with CJI had the lowest (55·4%). Admissions for persons experiencing both homelessness and CJI disproportionally occurred in the West (47·9%) and Midwest (24·9%) US census regions compared with those experiencing neither (15·4% and 18·2%, respectively).

**Table 1:** Sociodemographic characteristics and treatment setting by group: homelessness, criminal justice involvement (CJI), both, and neither.

	Group			
	Homelessness n=2,524,413 (15·1%)	CJI n=4,764,750 (28·4%)	Both n=509,902 (3·0%)	Neither n=8,950,797 (53·4%)
Age				
18-24	230,886 (9·2%)	1,137,667 (23·9%)	73,841 (14·5%)	1,358,732 (15·2%)
25-29	319,273 (12·7%)	905,912 (19·0%)	77,718 (15·2%)	1,508,724 (16·9%)
30-39	650,572 (25·8%)	1,265,755 (26·6%)	136,790 (26·8%)	2,451,878 (27·4%)
40-49	760,125 (30·1%)	908,888 (19·1%)	129,582 (25·4%)	2,063,002 (23·1%)
50+	563,557 (22·3%)	546,528 (11·5%)	91,971 (18·0%)	1,568,461 (17·5%)
Male	1,857,119 (73·6%)	3,503,948 (73·6%)	370,712 (72·7%)	5,430,908 (60·7%)
Education				
Less than high school	820,989 (32·5%)	1,384,546 (29·1%)	175,678 (34·5%)	2,388,422 (26·7%)
High school/GED	1,143,530 (45·3%)	2,226,945 (46·7%)	236,518 (46·4%)	3,972,578 (44·4%)
Some college or higher	559,894 (22·2%)	1,153,259 (24·2%)	97,706 (19·2%)	2,589,797 (28·9%)
Race				
White	1,294,093 (51·3%)	2,921,550 (61·3%)	268,890 (52·7%)	5,870,149 (65·6%)
Black	682,889 (27·1%)	913,318 (19·2%)	99,638 (19·5%)	1,595,957 (17·8%)
Hispanic	151,546 (6·0%)	329,092 (6·9%)	38.423 (7·5%)	450,957 (5·0%)
American Indian/Native Hawaiian or Alaskan	66,417 (2·6%)	129,650 (2·7%)	33,640 (6·6%)	164,309 (1·8%)
Other	329,468 (13·1%)	471,140 (9·9%)	69,311 (13·6%)	869,448 (9·7%)
Unemployed	2,396,330 (94·9%)	2,638,854 (55·4%)	448,449 (88·0%)	6,645,338 (74·2%)
US Census Region				
Northeast	1,036,574 (41·1%)	1,355,694 (28·5%)	92,158 (18·1%)	3,749,156 (41·9%)
Midwest	361,926 (14·3%)	1,241,302 (26·1%)	127,081 (24·9%)	1,628,701 (18·2%)
South	413,117 (16·4%)	1,106,228 (23·2%)	46,336 (9·1%)	2,191,572 (24·5%)
West	712,796 (28·2%)	1,061,526 (22·3%)	244,327 (47·9%)	1,381,368 (15·4%)
Treatment Setting				
Detox	1,244,559 (49·3%)	324,381 (6·8%)	114,738 (22·5%)	2,297,919 (25·7%)
Residential	681,232 (27·0%)	392,616 (8·2%)	158,625 (31·1%)	1,444,661 (16·1%)
Ambulatory	598,622 (23·7%)	4,047,753 (85·0%)	236,539 (46·4%)	5,208,217 (58·2%)

Data are n (%). Percentages are provided as the percentage of the total number of admissions within a specific group with non-missing values for each variable.

### Substance use treatment trends

**Figure 3:**
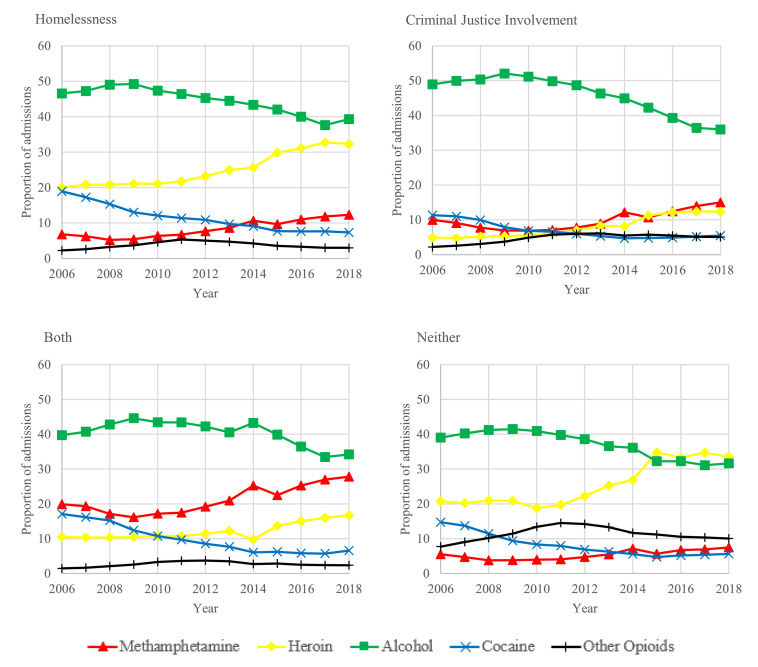
Adjusted trends in primary substance on admission by year and group: homelessness, criminal justice involvement (CJI), both, and neither, 2006-2018.

Between 2006 and 2018, substance use treatment admission trends varied substantially across groups experiencing homelessness, CJI, both, or neither. Alcohol use was the most common reason for treatment in 2006 but decreased between 14% and 27% in all groups by 2018. Similarly, the proportion of cocaine or crack-related treatment admissions decreased over 50% in all groups (Figure 3)*.*

In 2018, methamphetamine was the third most common reason for treatment admission among individuals experiencing homelessness only (12·3%; 95% CI: 12·2-12·4%) (appendix table 1). In 2018, methamphetamine was the second most common reason for treatment admission, after alcohol, among those experiencing CJI only (15·0%; 95% CI: 14·9-15·1%) as well as both homelessness and CJI (27·8%; 95% CI: 27·4-28·2%) representing a 50% and 40% increase for both groups, respectively. Among individuals experiencing homelessness, the largest relative shift was an 80% increase in the proportion of methamphetamine-related admissions.

Among individuals experiencing neither homelessness nor CJI, the largest relative shift was a 62% increase in the proportion of heroin-related admissions. By 2018, heroin was the most common reason for treatment admission among individuals experiencing neither homelessness nor CJI (33·6%; 95% CI: 33·4-33·7%). Similarly, the proportion of treatment admissions for “other opioids” rose 30% and was the third most common reason for admission among individuals experiencing neither homelessness nor CJI (10·0%; 95% CI: 10·0-10·1%) in 2018. Conversely, “other opioids” remained the least common reason for treatment admission among individuals experiencing homelessness (3·0%; 95% CI: 3·0-3·1%), CJI (5·1%; 95% CI: 5·0-5·2%), or both (2·3%; 95% CI: 2·2-2·5%) in 2018. For each group the linear trends were significant for all substances (P<0.001), except for the proportion of other opioid-involved treatment admissions among individuals experiencing homelessness only (P=0.053) (appendix table 1).

**Figure 4:**
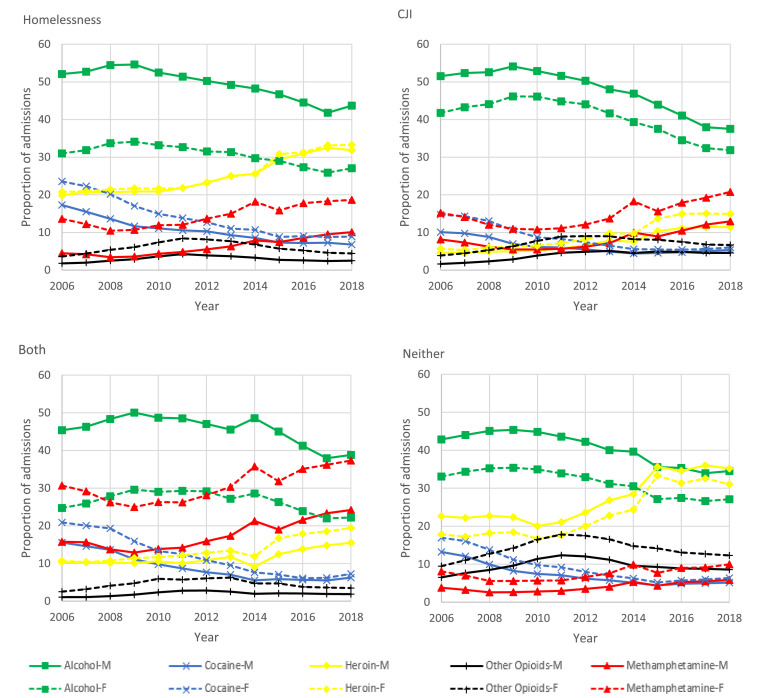
Adjusted trends in primary substance on admission by year, sex, and group: homelessness, criminal justice involvement (CJI), both, and neither, 2006-2018.

Among all groups the proportion of treatment admissions for alcohol was higher among men, while the proportion of admissions for cocaine or crack, heroin, other opiates, and methamphetamine was higher among women. The proportion of treatment admissions for alcohol was most similar among men (37·5%; 95% CI: 37·3-37·7%) and women (31·9%; 95% CI: 31·6-32·2%) experiencing CJI in 2018. However, treatment admissions for alcohol were substantially more common among men than women for individuals experiencing homelessness (43·7%; 95% CI: 43·5-43·9% vs. 27·0%; 95% CI: 26·7-27·4%) and both homelessness and CJI (38·8%; 95% CI: 38·4-39·3% vs. 22·2%; 95% CI: 21·5-22·9%). Among women experiencing homelessness and both homelessness and CJI treatment admissions were most commonly for heroin (33·3%; 95% CI: 32·9-33·6%) and methamphetamine (37·3%; 95% CI: 36·5-38·1%), respectively (Figure 4).

**Figure 5:**
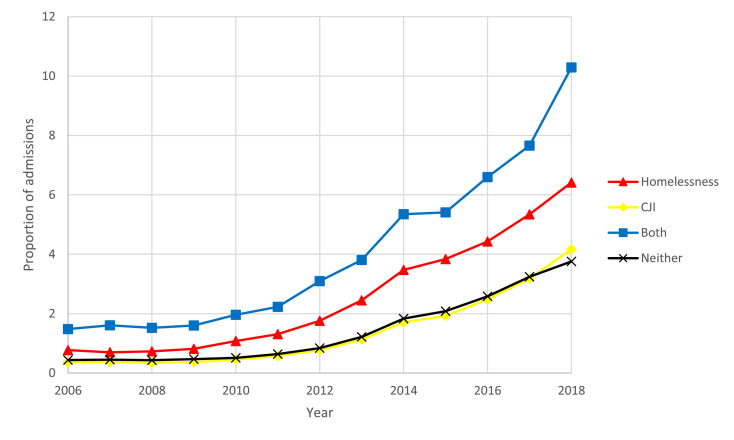
Proportion of admissions for both methamphetamine and heroin use by year and group: homelessness, criminal justice involvement (CJI), both, and neither, 2006-2018.

Between 2006 and 2018, all groups experienced a substantial increase, ranging from 595% to 1049%, in the proportion of treatment admissions related to both heroin and methamphetamine. Throughout the study period, individuals experiencing both CJI and homelessness had the highest proportion of treatment admissions related to both heroin and methamphetamine. In 2018, 10·3% (95% CI: 10·0-10·6%) of treatment admissions for this group contained both heroin and methamphetamine compared to 6·4% (95% CI: 6·3-6·5%), 4·2% (95% CI: 4·1-4·2%), and 3·8% (95% CI: 3·7-3·8%) of treatment admissions for those experiencing homelessness only, CJI only, or neither, respectively (Figure 5).

### Treatment characteristics

There were significant differences in treatment characteristics between groups. Treatment admissions for individuals experiencing homelessness were most commonly at a detoxification facility (49·3%) while admissions for individuals experiencing CJI were the least common (6·8%). Conversely, treatment admissions for individuals with CJI were the most common in an ambulatory setting (85·0%), while admissions for those experiencing homelessness were the least common (23·7%). Treatment admissions for those experiencing both homelessness and CJI were the most likely to occur in a residential setting (31·1%) (Table 1).

With and without adjusting for treatment setting, prior substance use treatment was most common among admissions for individuals experiencing homelessness (70·3%; 95% CI: 70·2-70·4% and 68·2%; 95% CI: 68·2-68·3%, respectively) and least common for those with CJI (55·6%; 95% CI: 55·6-55·7% and 57·0%; 95% CI: 56·9-57·0%, respectively) (Table 2). Among admissions with heroin or other opioids as the primary substance, individuals experiencing homelessness, CJI, and both received substantially lower rates of medication for OUD (19·2%; 95% CI:19.1-19.3%, 10·6%; 95% CI:10.3-10.8%, and 8·3%; 95% CI:8.2-8.3%, respectively) than individuals experiencing neither (37.6·2%; 95% CI:37.6-37.7%). When treatment facility was included as a co-variate the rate of medication for OUD among treatment admissions for individuals experiencing homelessness increased but remained lower than the rate among treatment admissions for individuals experiencing neither (29·5%; 95% CI:29.4-29.7% vs. 36·4%; 95% CI:36.4-36.4%) (Table 2).

**Table 2:** Treatment characteristics by group: homelessness, criminal justice involvement (CJI), both, and neither.

	Group			
	Homelessness	CJI	Both	Neither
*Adjusted for sociodemographic characteristics only*				
Prior Treatment	70·3% (70·2%-70·4%)	55·6% (55·6%-55·7%)	63·7% (63·6%-63·9%)	62·9% (62·8%-62·9%)
Receipt of medication for opioid use disorder^a^	19·2% (19·1%-19·3%)	10·5% (10·4%-10·6%)	7·9% (7·7%-8·0%)	37·6% (37·6%-37·7%)
*Adjusted for sociodemographic characteristics and treatment setting*				
Prior Treatment-adjusted	68·2% (68·2%-68·3%)	57·0% (56·9%-57·0%)	62·3% (62·2%-62·5%)	62·8% (62·8%-62·8%)
Receipt of medication for opioid use disorder-adjusted^a^	29·5% (29·4%-29·7%)	10·6% (10·3%-10·8%)	8·3% (8·2%-8·3%)	36·4% (36·4%-36·4%)

Data are % (95% CI). All proportions were estimated with margins from logistic regression controlling for demographics. All pairs were statistically significant at a level of p<0·001. ^a^Receipt of medication for opioid use disorder was estimated among admissions with heroin or other opioids listed as the primary substance.

In analyses to assess the impact of our cohort specifications, we included an additional 3,486,327 treatment admissions of dependently housed individuals in the groups not experiencing homelessness, generating a final sample of 20,237,811 treatment admissions. Sociodemographic characteristics and trends in overall treatment admissions did not differ substantively from our primary analysis (appendix figure 1). After including dependently housed individuals, a higher proportion of admissions in 2018 among individuals with CJI were for methamphetamine use (18·4%; 95% CI: 18·3-18·5% vs. 15·0%; 95% CI: 14·9-15·1%) and for both methamphetamine and heroin use (5·6%; 95% CI: 5·5-5·6% vs. 4·2%; 95% CI: 4·1-4·2%) compared with our primary analysis (appendix figures 2 and 3). In a sensitivity analysis restricted to treatment admissions of individuals without prior treatment, the general trends in treatment utilization did not substantively differ from the primary analysis (appendix figure 5). Compared to the primary analysis, the proportion of admissions for heroin-related treatment in this sensitivity analysis was lower, suggesting individuals may receive treatment for heroin use more often.

## Discussion

In our analysis of US substance use treatment admissions between 2006 and 2018, we found important differences between trends in substance use treatment admissions for individuals experiencing homelessness only, CJI only, both homelessness and CJI, or neither. Methamphetamine-related treatment increased substantially among individuals experiencing homelessness, CJI, or both. This rise was particularly prevalent for individuals experiencing both homelessness and CJI; by 2018, 28% of treatment admissions for individuals experiencing both homelessness and CJI were for methamphetamine compared with 7·5% in the general population. These national trends may reflect local reports from the west coast of high rates of methamphetamine use among individuals experiencing homelessness.^41^ A higher proportion of treatment admissions were related to heroin for individuals experiencing neither homelessness nor CJI compared to admissions for individuals experiencing both homelessness and CJI as well as CJI alone. The divergence in treatment admission trends for individuals experiencing neither homelessness nor CJI and those experiencing one or both illustrates that the treatment needs of marginalized groups differ in important ways from the general population. While investments in opioid treatment will benefit all groups, a smaller proportion of individuals experiencing homelessness or CJI will be impacted because treatment admissions among these groups are disproportionately for substances other than opioids, particularly methamphetamine.

Our results also indicate that individuals experiencing homelessness, CJI, or both experience differences in treatment indicative of lower overall treatment quality. For example, treatment admissions for individuals experiencing homelessness were almost twice as likely to be to a detoxification facility and half as likely to an ambulatory setting than admissions for the general population. This pattern is problematic as the American Society of Addiction Medicine has stated detoxification alone is not appropriate treatment for opioid use disorder.^42^ Including treatment facility type as a co-variate accounted for a larger share of the difference in medications for OUD between individuals experiencing homelessness and individuals experiencing neither homelessness nor CJI relative to other characteristics. Disproportionate care at certain facility types explains some of the difference in receipt of medications for OUD between groups. Across all groups fewer than 40% of admissions primarily for treatment of heroin or other opioid use received medications for OUD. However, experiencing homelessness, CJI, or both was associated with notably lower rates of medications for OUD use. Admissions for those who experienced both homelessness and CJI had the lowest rate of medications for OUD use, four and a half fold lower than individuals experiencing neither. This finding builds on previous research that identified limited access to medications for OUD among individuals with CJI to show that those experiencing both homelessness and CJI have even lower rates of medications for OUD use.^16^ Importantly, homelessness, CJI, or both are not contraindications for medications for OUD.

A rise in treatment admissions for concurrent methamphetamine and heroin use in the general population has previously been described.^43^ Our results show that the proportion of treatment admissions for both methamphetamine and heroin use disproportionately rose among individuals experiencing homelessness or both homelessness and CJI. By 2018, use of both methamphetamine and heroin was indicated for 1 in 10 admissions for individuals experiencing both homelessness and CJI. Use of both methamphetamine and heroin has been shown to be associated with unstable housing, worse health outcomes, and lower rates of medication for OUD.^34^^,^^44^ Given low rates of medications for OUD use among admissions for those with both homelessness and CJI, future research and interventions should focus on the unique treatment needs of this population, particularly the rising rates of admissions for concurrent methamphetamine and heroin use we found in this study, and barriers to initiation and continuation of evidence-based treatment programs.

Treatment admissions for men were more likely to be alcohol-related compared to women, among all four groups. This finding extends previous work that has documented lower treatment rates for alcohol use among women than men in the general population but similar overall treatment rates for other substance use disorders.^45^, ^46^, ^47^ The difference in alcohol treatment between men and women was most pronounced individuals experiencing homelessness or both homelessness and CJI compared to admissions for individuals experiencing neither. It is important to reduce the intersecting stigmas of gender, drug use, incarceration, and homelessness which may limit access and quality of treatment services.^48^ Treatment facilities should be equipped to serve the unique needs of individuals experiencing homelessness and/or CJI. To provide patient-centered care it may be important to consider the different pathways to homelessness and/or CJI. For example, men were more likely to report discharge from an institution or substance use as a reason for homelessness while women were more likely to report eviction or interpersonal conflict.^49^^,^^50^ An understanding of these pathways, and the differing trends in substance use treatment admissions between genders, can help ensure treatment facilities are equipped to treat different substance use disorders and address unique external factors for men and women.

In this paper, we show that trends in substances leading to treatment admission differ between people experiencing homelessness, CJI, both or neither. Importantly, this suggests that systems structured around treatment patterns in the general population may not adequately meet the needs of individuals who experiences homelessness and/or CJI. For example, there is potential for underinvestment in methamphetamine-related treatment because of its lower use in the general population, though such treatment is highly prevalent among people experiencing homelessness or incarceration. In addition to building capacity for treatment admission types that occur disproportionately among socioeconomically excluded populations, treatment programs could consider other co-occurring conditions and structural barriers for people who experience homelessness or incarceration.^51^ Individuals experiencing homelessness or CJI have substantially higher rates of mental illness and co-existing physical health conditions like hepatitis C and HIV compared to the general population.^13^^,^^17^^,^^52^, ^53^, ^54^, ^55^, ^56^ Using a syndemic approach that addresses multiple co-occurring disease conditions and environmental/socioeconomic factors, treatment programs could invest in co-located services that meet intersecting health needs.^57^^,^^58^

This study has important limitations. Although TEDS-A is the most comprehensive substance use treatment data set in the US, it does not necessarily include information from facilities which do not receive public funding. Therefore, changes we describe among admissions within community treatment facilities that receive public funding may not necessarily be extrapolated to other treatment settings such as jails or prisons. However, data from the National Survey on Drug Use and Health from 2015-2019 indicates that 87% of individuals who received substance use treatment in a jail or prison also received treatment in a community setting within the past year.^59^ Additionally, because TEDS-A only records information on housing status or referral source on admission we are unable to identify individuals with more distant homelessness and/or CJI. However, this potential misspecification would bias our results by decreasing the total admissions indicating homelessness and/or CJI as well as the observed differences between these groups and the general population. Accordingly, the total admissions and differences we observe between groups can be considered conservative estimates. Other factors such as health insurance coverage and co-morbid diagnosis may affect substance use treatment utilization, however due to data limitations we were unable to adjust for them in our estimates of treatment. Finally, because the TEDS-A includes data at the treatment admission level, rather than individual level, the changes in treatment are not necessarily indicative of underlying changes in the pattern of substance use. Future research should explore whether the trends we describe for socioeconomically excluded groups, differ by demographic characteristics such as age, sex, race and ethnicity.

Between 2006 and 2018, reasons for admission to treatment differed substantially between individuals experiencing homelessness, CJI, both or neither. Methamphetamine-related admissions increased to a greater degree for individuals experiencing homelessness, CJI, or both compared to admissions for those who experience neither. Admissions with neither homelessness nor CJI had a larger increase in the proportion related to heroin-use. Rates of medication for OUD use were also lower for groups experiencing homelessness, CJI, or both. To better serve populations experiencing homelessness, CJI, or both it is important that community treatment facilities are equipped to appropriately treat methamphetamine and polysubstance use among individuals with these complex social factors.

## Declaration of interests

RDS was supported by NIH MSTP grant T32 GM008244. KDV was supported by the National Institute of Diabetes and Digestive and Kidney Diseases of the National Institutes of Health under Award Number K23DK118117. TNAW was supported by the White Paper Scholars Career Development Award – Hennepin Healthcare. The content is solely the responsibility of the authors and does not necessarily represent the official views of the National Institutes of Health.

## Appendix Supplementary materials

Image, application 1
