# Supplementary material for: A longitudinal cross-sectional analysis of substance use treatment trends for individuals experiencing homelessness, criminal justice involvement, both, or neither - United States, 2006-2018
Source: Lancet Reg Health Am. 2022 Jan 6;7:100174. doi: 10.1016/j.lana.2021.100174 (PMC8979492; doi:10.1016/j.lana.2021.100174)
Supplement: Supplementary file 1 [file mmc1.docx]

**Appendix Tables**

**Appendix Table 1** Adjusted proportion in primary substance on admission by year and group: homelessness, criminal justice involvement (CJI), both, and neither, 2006-2018

|  |  | 2006 | 2007 | 2008 | 2009 | 2010 | 2011 | 2012 | 2013 | 2014 | 2015 | 2016 | 2017 | 2018 | Trend |
| --- | --- | --- | --- | --- | --- | --- | --- | --- | --- | --- | --- | --- | --- | --- | --- |
| Homeless | Methamphetamine | 6.8%  (6.7-6.9) | 6.3%  (6.2-6.4) | 5.2%  (5.1-5.3) | 5.4  (5.3-5.5) | 6.4%  (6.2-6.5) | 6.7%  (6.6-6.8) | 7.7%  (7.6-7.8) | 8.6%  (8.5-8.7) | 10.6%  (10.5-10.8) | 9.7%  (9.6-9.8) | 11.0%  (10.9-11.1) | 11.8%  (11.7-11.9) | 12.3%  (12.2-12.4) | P<0.001 |
|  | Heroin | 20.0%  (19.9-20.2) | 20.8%  (20.6-21.0) | 20.8%  (20.7-21.0) | 21.0%  (20.9-21.2) | 21.0%  (20.9-21.2) | 21.8%  (21.6-21.9) | 23.2%  (23.0-23.3) | 25.0%  (25.4-25.8) | 25.6%  (25.4-25.8) | 29.8%  (29.7-30.0) | 31.1%  (30.9-31.3) | 32.7%  (32.6-32.9) | 32.3%  (32.1-32.5) | P<0.001 |
|  | Alcohol | 46.6%  (46.4-46.8) | 47.2% (47.0-47.4) | 49.0% (48.8-49.2) | 49.2%  (49.0-49.4) | 47.4%  (47.2-47.6) | 46.4%  (46.2-46.6) | 45.3%  (45.1-45.5) | 44.5%  (44.3-44.7) | 43.4%  (43.2-43.6) | 42.0%  (41.8-42.2) | 40.0%  (39.8-40.2) | 37.6%  (37.4-37.8) | 39.3%  (39.1-39.5) | P<0.001 |
|  | Cocaine | 19.0%  (18.8-19.1) | 17.3%  (17.1-17.4) | 15.3%  (15.2-15.5) | 13.0%  (12.9-13.2) | 12.1%  (12.0-12.2) | 11.4%  (11.3-11.5) | 10.9%  (10.8-11.0) | 9.7%  (9.6-9.9) | 9.1%  (9.0-9.3) | 7.7%  (7.6-7.8) | 7.6%  (7.5-7.8) | 7.7%  (7.6-7.8) | 7.4%  (7.2-7.5) | P<0.001 |
|  | Other Opioids | 2.3%  (2.2-2.3) | 2.6%  (2.5-2.7) | 3.3%  (3.2-3.3) | 3.7%  (3.6-3.8) | 4.6%  (4.5-4.7) | 5.4%  (5.3-5.4) | 5.0%  (5.0-5.1) | 4.7%  (4.6-4.8) | 4.2%  (4.1-4.3) | 3.5%  (3.5-3.6) | 3.3%  (3.2-3.4) | 3.0%  (2.9-3.1) | 3.0%  (3.0-3.1) | P=0.053 |
|  | Methamphetamine & Heroin | 0.8%  (0.7-0.8) | 0.7%  (0.7-0.7) | 0.7%  (0.7-0.8) | 0.8%  (0.8-0.9) | 1.1%  (1.0-1.1) | 1.3%  (1.3-1.4) | 1.8%  (1.7-1.8) | 2.5%  (2.4-2.5) | 3.5%  (3.4-3.6) | 3.8%  (3.8-3.9) | 4.4%  (4.3-4.5) | 5.3%  (5.3-5.4) | 6.4%  (6.3-6.5) | P<0.001 |
| CJI | Methamphetamine | 10.0%  (9.9-10.1) | 9.1%  (9.0-9.2) | 7.8%  (7.7-7.8) | 6.9%  (6.9-7.0) | 6.9%  (6.8-7.0) | 7.1%  (7.0-7.2 | 7.8%  (7.7-7.9) | 8.9%  (8.8-9.0) | 12.2%  (12.0-12.3) | 10.7%  (10.6-10.8) | 12.5%  (12.3-12.6) | 14.0%  (13.9-14.1) | 15.0%  (14.9-15.1) | P<0.001 |
|  | Heroin | 4.9%  (4.8-5.0) | 4.8%  (4.7-4.8) | 5.2%  (5.1-5.3) | 5.3%  (5.3-5.4) | 5.6%  (5.5-5.6) | 6.1%  (6.0-6.2) | 7.0%  (6.9-7.1) | 8.3%  (8.2-8.4) | 8.2%  (8.1-8.2) | 11.3%  (11.2-11.4) | 12.2%  (12.1-12.3) | 12.4%  (12.3-12.5) | 12.4%  (12.3-12.5) | P<0.001 |
|  | Alcohol | 49.0%  (48.8-49.1) | 50.0% (49.8-50.1) | 50.3%  (50.2-50.5) | 52.1%  (51.9-52.2) | 51.2%  (51.0-51.3) | 49.9%  (49.7-50.0) | 48.7%  (48.5-48.8) | 46.3%  (46.2-46.5) | 44.9%  (44.7-45.1) | 42.2%  (42.1-42.4) | 39.3%  (39.1-39.5) | 36.5%  (36.3-36.6) | 36.0%  (35.8-36.1) | P<0.001 |
|  | Cocaine | 11.4%  (11.3-11.5) | 11.0%  (10.9-11.1) | 9.9%  (9.9-10.0) | 7.9%  (7.9-8.0) | 6.8%  (6.8-6.9) | 6.6%  (6.5-6.7) | 6.0%  (5.9-6.1) | 5.3%  (5.3-5.4) | 4.7%  (4.6-4.8) | 4.8%  (4.7-4.9) | 4.9%  (4.8-5.0) | 5.2%  (5.1-5.2) | 5.5%  (5.4-5.6) | P<0.001 |
|  | Other Opioids | 2.2%  (2.2-2.3) | 2.6%  (2.5-2.6) | 3.1%  (3.1-3.2) | 3.7%  (3.7-3.8) | 4.9%  (4.8-5.0) | 5.7%  (5.7-5.8) | 6.0%  (5.9-6.1) | 6.1%  (6.1-6.2) | 5.5%  (5.4-5.6) | 5.7%  (5.7-5.8) | 5.5%  (5.4-5.6) | 5.1%  (5.1-5.2) | 5.1%  (5.0-5.2) | P<0.001 |
|  | Methamphetamine & Heroin | 0.4%  (0.3-0.4) | 0.4%  (0.3-0.4) | 0.3%  (0.3-0.4) | 0.4%  (0.3-0.4) | 0.5%  (0.4-0.5) | 0.6%  (0.5-0.6) | 0.8%  (0.7-0.8) | 1.1%  (1.1-1.2) | 1.7%  (1.7-1.8) | 1.9%  (1.9-2.0) | 2.5%  (2.4-2.5) | 3.2%  (3.1-3.2) | 4.2%  (4.1-4.2) | P<0.001 |
| Both | Methamphetamine | 19.9%  (19.5-20.2) | 19.3%  (18.9-19.7) | 17.1%  (16.8-17.5) | 16.1%  (15.8-16.5) | 17.2%  (16.8-17.5) | 17.4%  (17.1-17.8) | 19.2%  (18.8-19.5) | 20.9%  (20.6-21.3) | 25.3%  (24.9-25.6) | 22.5%  (22.2-22.8) | 25.3%  (24.9-25.6) | 26.9%  (26.6-27.3) | 27.8%  (27.4-28.3) | P<0.001 |
|  | Heroin | 10.5%  (10.2-10.8) | 10.3%  (10.0-10.6) | 10.3%  (10.0-10.6) | 10.5%  (10.2-10.8) | 10.8%  (10.5-11.2) | 10.6%  (10.3-10.9) | 11.4%  (11.1-11.7) | 12.2%  (11.9-12.5) | 9.7%  (9.5-10.0) | 13.6%  (13.3-14.0) | 15.0%  (14.6-15.3) | 15.9%  (15.6-16.2) | 16.7%  (16.3-17.0) | P<0.001 |
|  | Alcohol | 39.7%  (39.3-40.2) | 40.7%  (40.3-41.2) | 42.8%  (42.3-43.2) | 44.6%  (44.1-45.0) | 43.4%  (43.0-43.9) | 43.4%  (42.9-43.8) | 42.2%  (41.8-42.6) | 40.5%  (40.1-40.9) | 43.3%  (42.8-43.7) | 39.9%  (39.5-40.3) | 36.5%  (36.1-36.9%) | 33.5%  (33.1-33.8) | 34.2%  (33.8-34.6) | P<0.001 |
|  | Cocaine | 17.1%  (16.7-17.4) | 16.1%  (15.8-16.5) | 15.2%  (14.9-15.5) | 12.4%  (12.1-12.7) | 10.7%  (10.4-11.0) | 9.7%  (9.4-10.0) | 8.5%  (8.2-8.8) | 7.7%  (7.4-7.9) | 6.1%  (5.8-6.3) | 6.2%  (6.0-6.4) | 5.8%  (5.6-6.0) | 5.7%  (5.5-5.9) | 6.6%  (6.3-6.8) | P<0.001 |
|  | Other Opioids | 1.5%  (1.3-1.6) | 1.7%  (1.5-1.8) | 2.1%  (2.0-2.2) | 2.5%  (2.4-2.7) | 3.3%  (3.1-3.5) | 3.6%  (3.4-3.8) | 3.7%  (3.5-3.9) | 3.5%  (3.3-3.7) | 2.7%  (2.6-2.8) | 2.8%  (2.7-3.0) | 2.5%  (2.4-2.7) | 2.4%  (2.3-2.5) | 2.3%  (2.2-2.5) | P<0.001 |
|  | Methamphetamine & Heroin | 1.5%  (1.4-1.6) | 1.6%  (1.5-1.7) | 1.5%  (1.4-1.6) | 1.6%  (1.5-1.7) | 2.0%  (1.8-2.1) | 2.2%  (2.1-2.4) | 3.1%  (2.9-3.3) | 3.8%  (3.6-4.0) | 5.4%  (5.1-5.6) | 5.4%  (5.2-5.6) | 6.6%  (6.4-6.8) | 7.7%  (7.5-7.9) | 10.3%  (10.0-10.6) | P<0.001 |
| Neither | Methamphetamine | 5.5%  (5.4-5.5) | 4.7%  (4.7-4.8) | 3.8%  (3.7-3.8) | 3.8%  (3.7-3.8) | 3.9%  (3.9-4.0) | 4.0%  (4.0-4.1) | 4.75  (4.7-4.8) | 5.4%  (5.4-5.5) | 7.1%  (7.0-7.2) | 5.7%  (5.6-5.7) | 6.7%  (6.7-6.8) | 6.9%  (6.9-7.0) | 7.5%  (7.4-7.5) | P<0.001 |
|  | Heroin | 20.7%  (20.6-20.8) | 20.2%  (20.1-20.3) | 20.9%  (20.8-21.0) | 20.8%  (20.7-20.9) | 18.8%  (18.7-18.9) | 19.7%  (19.6-19.8) | 22.2%  (22.1-22.3) | 25.2%  (25.1-25.3) | 26.9%  (26.8-27.0) | 34.%  (34.6-34.8) | 33.3%  (33.2-33.4) | 34.7%  (34.6-34.8) | 33.6%  (33.4-33.7) | P<0.001 |
|  | Alcohol | 39.0%  (38.9-39.1) | 40.2%  (40.1-40.3) | 41.2% (41.1-41.3) | 41.5% (41.3-41.6) | 40.9%  (40.8-41.1) | 39.8%  (39.7-39.9) | 38.5%  (38.4-38.7) | 36.5%  (36.4-36.7) | 36.1%  (36.0-36.2) | 32.2%  (32.1-32.3) | 32.2%  (32.1-32.4) | 31.1%  (31.0-31.2) | 31.6%  (31.5-31.7) | P<0.001 |
|  | Cocaine | 14.7%  (14.6-14.8) | 13.7%  (13.6-13.8) | 11.4%  (11.4-11.5) | 9.4%  (9.3-9.5) | 8.3%  (8.3-8.4) | 7.9%  (7.8-8.0) | 6.9%  (6.8-7.0) | 6.2%  (6.2-6.3) | 5.6%  (5.5-5.6) | 4.7%  (4.7-4.8) | 5.2%  (5.1-5.2) | 5.4%  (5.3-5.4) | 5.6%  (5.6-5.7) | P<0.001 |
|  | Other Opioids | 7.7%  (7.6-7.8) | 9.0%  (8.9-9.1) | 10.2%  (10.1-10.2) | 11.4%  (11.4-11.5) | 13.4%  (13.3-13.5) | 14.5% (14.4-14.6) | 14.2%  (14.1-14.3) | 13.3%  (13.2-13.4) | 11.6%  (11.5-11.7) | 11.2%  (11.1-11.3) | 10.5%  (10.5-10.6) | 10.3%  (10.3-10.4) | 10.0%  (10.0-10.1) | P<0.001 |
|  | Methamphetamine & Heroin | 0.4%  (0.4-0.5) | 0.5%  (0.4-0.5) | 0.4%  (0.4-0.4) | 0.5%  (0.5-0.5) | 0.5%  (0.5-0.5) | 0.6%  (0.6-0.7) | 0.8%  (0.8-0.9) | 1.2%  (1.2-1.2) | 1.8%  (1.8-1.9) | 2.1%  (2.0-2.1) | 2.6%  (2.5-2.6) | 3.2%  (3.2-3.3) | 3.8%  (3.7-3.8) | P<0.001 |

Data are % (95% CI). All proportions were estimated with margins from logistic regression controlling for demographics and treatment facility setting. Trend significance was assessed using year as a continuous variable.

**Appendix Table 2** Sociodemographic characteristics by group: homelessness, criminal justice involvement (CJI), both, and neither

|  | **Group** | | | |
| --- | --- | --- | --- | --- |
|  | **Homelessness**  n=2,524,413 (12·5%) | **CJI^a^**  n=6,200,531 (30·6%) | **Both**  n=509,902 (2·5%) | **Neither^a^**  n=11,000,994 (54·4%) |
| Age |  |  |  |  |
| 18-24 | 230,886 (9·2%) | 1,551,968 (25·0%) | 73,841 (14·5%) | 1,776,918 (16·2%) |
| 25-29 | 319,273 (12·7%) | 1,186,688 (19·1%) | 77,718 (15·2%) | 1,867,531 (17·0%) |
| 30-39 | 650,572 (25·8%) | 1,643,863 (26·5%) | 136,790 (26·8%) | 2,991,529 (27·2%) |
| 40-49 | 760,125 (30·1%) | 1,158,545 (18·7%) | 129,582 (25·4%) | 2,499,803 (22·7%) |
| 50+ | 563,557 (22·3%) | 659,467 (10·6%) | 91,971 (18·0%) | 1,865,213 (17·0%) |
| Male | 1,857,119 (73·6%) | 4,544,561 (73·3%) | 370,712 (72·7%) | 6,698,209 (60·9%) |
| Education |  |  |  |  |
| Less than high school | 820,989 (32·5%) | 1,892,732 (30·5%) | 175,678 (34·5%) | 3,009,964 (27·4%) |
| High school/GED | 1,143,530 (45·3%) | 2,900,403 (46·8%) | 236,518 (46·4%) | 4,911,612 (44·7%) |
| Some college or higher | 559,894 (22·2%) | 1,407,396 (22·7%) | 97,706 (19·2%) | 3,079,418 (28·0%) |
| Race |  |  |  |  |
| White | 1,294,093 (51·3%) | 3,705,521 (59·8%) | 268,890 (52·7%) | 7,052,672 (64·1%) |
| Black | 682,889 (27·1%) | 1,179,211 (19·0%) | 99,638 (19·5%) | 2,017,078 (18·3%) |
| Hispanic | 151,546 (6·0%) | 451,092 (7·3%) | 38.423 (7·5%) | 593,108 (5·4%) |
| American Indian/Native Hawaiian or Alaskan | 66,417 (2·6%) | 182,049 (2·9%) | 33,640 (6·6%) | 212,063 (1·9%) |
| Other | 329,468 (13·1%) | 682,658 (11·0%) | 69,311 (13·6%) | 1,126,073 (10·2%) |
| Unemployed | 2,396,330 (94·9%) | 3,756,449 (60·6%) | 448,449 (88·0%) | 8,433,554 (76·7%) |
| US Census Region |  |  |  |  |
| Northeast | 1,036,574 (41·1%) | 1,582,194 (25·5%) | 92,158 (18·1%) | 4,244,105 (38·6%) |
| Midwest | 361,926 (14·3%) | 1,631,978 (26·3%) | 127,081 (24·9%) | 2,146,597 (19·5%) |
| South | 413,117 (16·4%) | 1,349,029 (21·8%) | 46,336 (9·1%) | 2,632,731 (23·9%) |
| West | 712,796 (28·2%) | 1,638,330 (26·4%) | 244,327 (47·9%) | 1,977,561 (18·0%) |
| Treatment Setting |  |  |  |  |
| Detox | 1,244,559 (49·3%) | 385,691 (6·82) | 114,738 (22·5%) | 2,667,852 (24·3%) |
| Residential | 681,232 (27·0%) | 734,003 (11·8%) | 158,625 (31·1%) | 1,982,901 (18·0%) |
| Ambulatory | 598,622 (23·7%) | 5,080,837 (81·9%) | 236,539 (46·4%) | 6,350,241 (57·7%) |

Data are n (%). Percentages are provided as the percentage of the total number of admissions within a specific group with non-missing values for each variable. ^a^The treatment groups with criminal justice involvement and neither homelessness nor criminal justice involvement include dependently housed individuals.

**Appendix Table 3** Treatment Characteristics by Group: Homeless, Criminal Justice Involvement (CJI), Both, and Neither

|  | **Group** | | | |
| --- | --- | --- | --- | --- |
|  | **Homelessness** | **CJI^a^** | **Both** | **Neither^a^** |
| *Adjusted for sociodemographic characteristics only* |  |  |  |  |
| Prior Treatment | 70·0% (69·9%-70·0%) | 57·0% (57·0%-57·1%) | 63·3% (63·2%-63·4%) | 63·2% (63·1%-63·2%) |
| Receipt of medication for opioid use disorder^b^ | 19·4% (19·3%-19·5%) | 9·3% (9·2%-9·3%) | 8·0% (7·8%-8·1%) | 36·9% (36·9%-37·0%) |
|  |  |  |  |  |
| *Adjusted for sociodemographic characteristics and treatment setting* |  |  |  |  |
| Prior Treatment-adjusted | 68·2% (68·2%-68·3%) | 58·0% (57·9%-58·0%) | 62·0% (61·9%-62·2%) | 63·1% (63·0%-63·1%) |
| Receipt of medication for opioid use disorder-adjusted^b^ | 29·6% (29·5%-29·7%) | 7·8% (7·8%-7·9%) | 10·7% (10·4%-10·9%) | 36·0% (35·9%-36·0%) |

Data are % (95% CI). Proportions were estimated with margins from logistic regression controlling for demographics. All pairs were statistically at a level of p<0·001, except for Prior Treatment percent between groups experiencing both andr neither which was not statistically different p=0·078 when adjusting for sociodemographic characteristics only. ^a^The groups with criminal justice involvement and neither homelessness nor criminal justice involvement include dependently housed individuals. ^b^Receipt of medication for opioid use disorder was estimated among admissions with heroin or other opioids listed as the primary substance.

**Appendix Table 4** Sociodemographic characteristics among individuals with no prior treatment by group: homelessness, criminal justice involvement (CJI), both, and neither

|  | **Group** | | | |
| --- | --- | --- | --- | --- |
|  | **Homelessness**  n=1,011,469 (13·3%) | **CJI**  n=2,437,958 (32·0%) | **Both**  n=199,220 (2·6%) | **Neither**  n=3,961,117 (52·1%) |
| Age |  |  |  |  |
| 18-24 | 99,974 (9·9%) | 676,420 (27·8%) | 35,083 (17·6%) | 694,782 (17·5%) |
| 25-29 | 122,160 (12·1%) | 462,631 (19·0%) | 31,335 (15·7%) | 676,425 (17·1%) |
| 30-39 | 254,759 (25·2%) | 605,815 (24·9%) | 51,923 (26·1%) | 1,050,462 (26·5%) |
| 40-49 | 313,163 (31·0%) | 422,945 (17·4%) | 47,099 (23·6%) | 870,068 (22·0%) |
| 50+ | 221,413 (21·9%) | 270,147 (11·1%) | 33,779 (17·0%) | 669,380 (16·9%) |
| Male | 759,677 (75·1%) | 1,798,871 (73·8%) | 146,862 (73·7%) | 2,403,752 (60·7%) |
| Education |  |  |  |  |
| Less than high school | 358,363 (35·4%) | 694,376 (30·5%) | 69,952 (35·1%) | 1,068,592 (27·0%) |
| High school/GED | 447,350 (44·2%) | 1,120,054 (45·9%) | 92,031 (46·2%) | 1,760,287 (44·4%) |
| Some college or higher | 205,756 (20·3%) | 623,528 (25·6%) | 37,237 (18·7%) | 1,132,238 (28·6%) |
| Race |  |  |  |  |
| White | 447,640 (44·2%) | 1,457,714 (59·8%) | 101,652 (51·0%) | 2,506,768 (63·3%) |
| Black | 330,220 (32·7%) | 468,939 (19·2%) | 42,039 (21·1%) | 741,886 (18·7%) |
| Hispanic | 53,196 (5·3%) | 188,411 (7·7%) | 14,848 (7·5%) | 207,414 (5·2%) |
| American Indian/Native Hawaiian or Alaskan | 17,821 (1·8%) | 56,364 (2·3%) | 9,925 (5·0%) | 66,273 (1·7%) |
| Other | 162,592 (16·1%) | 266,530 (10·9%) | 30,756 (15·4%) | 438,776 (11·1%) |
| Unemployed | 960,470 (95·0%) | 1,224,295 (50·2%) | 171,901 (86·3%) | 2,823,945 (71·3%) |
| US Census Region |  |  |  |  |
| Northeast | 468,793 (46·4%) | 570,312 (23·4%) | 29,662 (14·9%) | 1,487,533 (37·6%) |
| Midwest | 100,285 (9·9%) | 584,072 (24·0%) | 50,188 (25·2%) | 618,369 (15·6%) |
| South | 191,603 (18·9%) | 687,332 (28·2%) | 24,379 (12·2%) | 1,196,157 (30·2%) |
| West | 250,788 (24·8%) | 596,242 (24·5%) | 94,991 (47·7%) | 659,058 (16·6%) |
| Treatment Setting |  |  |  |  |
| Detox | 613,864 (60·7%) | 194,131 (8·0%) | 51,168 (25·7%) | 1,250,766 (31·6%) |
| Residential | 179,409 (17·7%) | 132,413 (5·4%) | 53,149 (24·7%) | 449,671 (11·4%) |
| Ambulatory | 218,196 (21·6%) | 2,111,414 (86·6%) | 94,903 (47·6%) | 2,260,680 (57·1%) |

Data are n (%). Percentages are provided as the percentage of the total number of admissions within a specific group with non-missing values for each variable.

**Appendix Table 5** Treatment Characteristics for individuals with no prior treatment by Group: Homeless, Criminal Justice Involvement (CJI), Both, and Neither

|  | **Group** | | | | |
| --- | --- | --- | --- | --- | --- |
|  | | **Homelessness** | **CJI** | **Both** | **Neither** |
| *Adjusted for sociodemographic characteristics only* | |  |  |  |  |
| Receipt of medication for opioid use disorder^a^ | | 12·4% (12·2%-12·5%) | 6·0% (5·9%-6·1%) | 3·9% (3·7%-4·1%) | 28·7% (28·6%-28·8%) |
|  | |  |  |  |  |
| *Adjusted for sociodemographic characteristics and treatment setting* | |  |  |  |  |
| Receipt of medication for opioid use disorder-adjusted^a^ | | 20·9% (20·7%-21·1%) | 4·6% (4·5%-4·7%) | 5·4% (5·1%-5·7%) | 27·8% (27·8%-27·8%) |

Data are % (95% CI). Proportions were estimated with margins from logistic regression controlling for demographics. All pairs were statistically at a level of p<0·001. ^a^Receipt of medication for opioid use disorder was estimated among admissions with heroin or other opioids listed as the primary substance.

**Appendix Figure Legends**

**Appendix Figure 1:** Trends in overall treatment admissions by year and group (including dependently housed individuals): homelessness, criminal justice involvement (CJI), both, and neither, 2006-2018

**Appendix Figure 2:** Adjusted trends in primary substance on admission by year and group (includes dependently housed individuals): homelessness, criminal justice involvement, both, and neither

**Appendix Figure 3:** Proportion of admissions for both methamphetamine and heroin use by year and group (including dependently housed individuals): homelessness, criminal justice involvement (CJI), both, and neither, 2006-2018

**Appendix Figure 4:** Trends in overall treatment admissions among individuals with no prior treatment by year and group: homelessness, criminal justice involvement (CJI), both, and neither, 2006-2018

**Appendix Figure 5:** Adjusted trends in primary substance on admission among individuals with no prior treatment by year and group homelessness, criminal justice involvement, both, and neither

**Appendix Figure 6:** Proportion of admissions for both methamphetamine and heroin use among individuals with no prior treatment by year and group: homelessness, criminal justice involvement (CJI), both, and neither, 2006-2018
